# A Mini Review on Carbon Quantum Dots: Preparation, Properties, and Electrocatalytic Application

**DOI:** 10.3389/fchem.2019.00671

**Published:** 2019-10-04

**Authors:** Xiao Wang, Yongqiang Feng, Peipei Dong, Jianfeng Huang

**Affiliations:** Shaanxi Key Laboratory of Green Preparation and Functionalization for Inorganic Materials, Key Laboratory of Auxiliary Chemistry and Technology for Chemical Industry, School of Materials Science and Engineering, Ministry of Education, Shaanxi University of Science and Technology, Xi'an, China

**Keywords:** carbon quantum dots, synthetic method, photoluminescence, electrocatalyst, electron transfer

## Abstract

Luminescent carbon quantum dots (CQDs) represent a new form of nanocarbon materials which have gained widespread attention in recent years, especially in chemical sensor, bioimaging, nanomedicine, solar cells, light-emitting diode (LED), and electrocatalysis. CQDs can be prepared simply and inexpensively by multiple techniques, such as the arc-discharge method, microwave pyrolysis, hydrothermal method, and electrochemical synthesis. CQDs show excellent physical and chemical properties like high crystallization, good dispersibility, photoluminescence properties. In particular, the small size, superconductivity, and rapid electron transfer of CQDs endow the CQDs-based composite with improved electric conductivity and catalytic activity. Besides, CQDs have abundant functional groups on the surface which could facilitate the preparation of multi-component electrical active catalysts. The interactions inside these multi-component catalysts may further enhance the catalytic performance by promoting charge transfer which plays an important role in electrochemistry. Most recent researches on CQDs have focused on their fluorescence characteristics and photocatalytic properties. This review will summarize the primary advances of CQDs in the synthetic methods, excellent physical and electronic properties, and application in electrocatalysis, including oxygen reduction reaction (ORR), oxygen evolution reaction (OER), hydrogen evolution reduction (HER), and CO_2_ reduction reaction (CO_2_RR).

## Introduction

In recent years, carbon-based nanomaterials such as carbon nanotubes (CNTs) (Rao et al., 2018), fullerenes (Lin et al., 2018), graphene (Clancy et al., 2018), and nanodiamonds (Georgakilas et al., 2015) have attracted a wide spread attention. However, the preparation and separation of nanodiamond are difficult; CNTs, fullerenes and graphene have poor water solubility and difficulty in providing strong fluorescence in visible areas, which greatly limits their application. Carbon quantum dots (CQDs) (Semeniuk et al., 2019) are novel zero-dimensional carbon-based nanomaterials known for their small size and relatively strong fluorescence characteristics. In the research field of CQDs, graphene quantum dots (GQDs), carbon nanodots (CNDs), and polymer dots (PDs) are the main research objects (Zhu et al., 2015). In some cases, CQDs are also called carbon dots (CDs). GQDs, CNDs, and PDs have similar size and photoelectrochemical properties, but they differ in the internal structure and chemical groups on the surface. They are monodisperse spherical nanoparticles with a carbon-based skeleton and a large amount of oxygen-containing groups on the surface (Lim et al., 2015). To make these materials fluorescent, their size and surface chemical groups must be carefully adjusted in order to finely tune the electronic structures. CQDs not only inherit the excellent optical properties of traditional semiconductor quantum dots, but also compensate for the deficiencies of the traditional materials in terms of cytotoxicity, environmental, and biohazard. In addition, CQDs are also featured with good water solubility, chemical stability, and photobleaching resistance, ease of surface functionalization and large-scale preparation (Yang et al., 2014). Since its discovery by researchers in 2004 first (Xu et al., 2004), it has been widely concerned by researchers in many fields such as biology, chemical sensing, nanomedicine and photoelectrocatalysis (Yuan et al., 2016, 2019; Sun and Lei, 2017). As of now, a lot of important progress in the synthesis and application of CQDs have been achieved. In this review, the synthetic methods and physical/chemical properties of these luminescent CQDs will be introduced firstly. Though the electrocatalytic properties of CQDs have been studied in recent years, there are no specific reviews that focus on the applications of CQDs in electrocatalysis aspect up to date. Thus, then we discuss in great detail on the applications of CQDs in several electrocatalytic reactions, including oxygen reduction reaction (ORR), oxygen evolution reaction (OER), hydrogen evolution reduction (HER), and CO_2_ reduction reaction (CO_2_RR), especially the advantages they could bring to these aspects.

## Synthetic Methods

**Figure 1:**
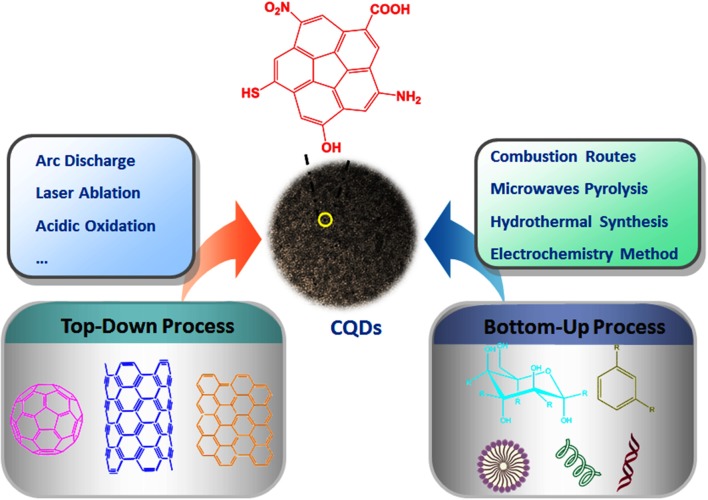
The typical approaches for the synthesis of CQDs.

Since the discovery of CQDs, a large variety of techniques for the preparation of CQDs have been developed (Mosconi et al., 2015; Lu and Yang, 2017; Wu et al., 2017; Yu et al., 2018; Anwar et al., 2019; Naik et al., 2019). Generally, synthetic methods of CQDs can be clarified into two groups: top-down and bottom-up methods (Figure 1). In top-down process, the macromolecule is destroyed or dispersed into small-sized CQDs by physical or chemical methods; while the bottom-up approach mainly refers to the polymerization and carbonization of a series of small molecules into CQDs through chemical reaction.

### Arc Discharge

Arora and Sharma (2014) indicated that arc discharge (Yatom et al., 2017) is a method to reorganize the carbon atoms decomposed from the bulk carbon precursors in the anodic electrode driven by the gas plasma generated in a sealed reactor. The temperature in the reactor can reach as high as 4,000 K under electric current in order to produce a high-energy plasma. In the cathode the carbon vapor assembly to form CQDs. The preparation of CQDs by arc discharge method was originated in 2004 (Xu et al., 2004). Xu et al. obtained three kinds of carbon nanoparticles with different relative molecular mass and fluorescence properties accidentally when preparing single-walled carbon nanotubes (SWNTs) by arc discharge method. The as-prepared CQDs can emit blue-green, yellow, and orange fluorescence at 365 nm. Further experiment demonstrated that the surface of CQDs was attached by hydrophilic carboxyl group. The CQDs obtained by this method have good water solubility, however, in general they possess a large particle size distribution in view of different sizes of carbon particles are formed during the discharge process. The large particle size would extensively decrease the specific surface area of CQDs, which may limit the active reaction sites during the electrocatalytic process.

### Laser Ablation

The laser ablation method (Kuzmin et al., 2010; Liu et al., 2015; Xiao et al., 2017; Donate-Buendia et al., 2018) uses a high-energy laser pulse to irradiate the surface of the target to a thermodynamic state in which high temperature and high pressure are generated, rapidly heats up and evaporates into a plasma state, and then the vapor crystallizes to form nanoparticles (Sun et al., 2006). Li et al. (2011) reported a facile approach to synthesize CQDs via laser irradiation of carbon precursor, which was dispersed in different ordinary organic solvents. The as-obtained CQDs exhibited visible and tunable photoluminescence (PL). Furthermore, Hu et al. (2009) demonstrated the surface state of the CQDs can be modified by selecting proper organic solvent during the laser irradiation process in order to tune the PL properties of the synthesized CQDs. Laser ablation is an effective method to prepare CQDs with narrow size distribution, good water solubility, and fluorescence characteristics. However, its complicated operation and high cost limit its application.

### Acidic Oxidation

Acid oxidation treatment have been widely used to exfoliate and decompose bulk carbon into nanoparticles, and simultaneously introduce hydrophilic groups, e.g., hydroxyl group or carboxyl group on the surface thereof to obtain CQDs (Shen and Xia, 2014; Zhang et al., 2017b), which could significantly improve the water solubility and fluorescence characteristics. In 2014, Yang et al. (2014) reported a large-scale synthesis of heteroatom-doped CQDs via acid oxidation, followed by hydrothermal reduction. Firstly, carbon nanoparticles derived from Chinese ink was oxidized by a mixture solution of HNO_3_, H_2_SO_4_, and NaClO_3_. Then the oxidized CQDs were hydrothermally reacted with dimethylformamide (DMF), sodium hydrosulfide (NaHS), and sodium selenide (NaHSe) as nitrogen source, sulfur source and selenium source, separately. The obtained N-CQDs, S-CQDs, and Se-CQDs exhibited tunable PL performance, higher quantum yield (QY), and longer fluorescence lifetime than the pure CQDs. Experimental results disclosed that the heavy-doped heteroatoms can affect the PL properties, which is positively related to the electronegativity of N, S, and Se. The active heteroatoms on the surface of CQDs would adjust the electronic structure of the corresponding CQDs and therefore would enable good electrocatalytic activity when used as electrocatalysts. On the other hand, as demonstrated in this work, such heavy-doped CQDs have the ability to coordinate with transition metal ions, the N-CQDs, S-CQDs, and Se-CQDs may also have the potential to absorb other metal ions, such as Fe^3+^, Co^2+^, and Ni^2+^, to form the so-called single-atom catalysts (SAC).

### Combustion/Thermal Routes

Recently, there has been much interest in developing bottom-up strategies for the synthesis of CQDs due to the facile procedure, ease of scale-up production, precise controllable design of initial molecules, low cost, and environmental benign operation (Li et al., 2012a; Guo et al., 2016; Thoda et al., 2018). The combustion/thermal oxidation method for the preparation of CQDs was first proposed by Xu et al. and followed by many researchers. For instance, Li et al. (2017) prepared a fluorescent GQDs by combustion of citric acid followed by functionalization with carboxyl groups through conjugation of acetic acid moieties under high temperature. The obtained GQDs possessed a uniform particle size of 8.5 nm and rich carboxyl groups on the surface of GQDs. Such oxygen-containing moieties would facilitate the adsorption of water molecules, which is beneficial to the electrocatalytic process in aqueous solution.

### Microwave Pyrolysis

Among the bottom-up approaches, the microwave pyrolysis method has been well-established due to the rapid synthesis and commercialization (Schwenke et al., 2015; In et al., 2017; Rai et al., 2017; Jiang et al., 2018; Shen et al., 2018). Zhu et al. reported a facile microwave pyrolysis approach to synthesize CQDs by combining poly(ethylene glycol) (PEG200) and a saccharide (glucose, fructose, etc.) in water to form a transparent solution, followed by heating in a microwave oven (Zhu et al., 2009). The obtained CQDs exhibited an excitation-dependent PL properties. This is a simple, fast and environment-friendly preparation method for CQDs rich in oxygen-containing groups, which would become the coordination sites of metal ions for the design of carbon-based electrocatalysts.

### Hydrothermal/Solvothermal Synthesis

In particular, hydrothermal method is one of the most commonly used procedure in CQDs synthesis (Shen et al., 2012; Lu et al., 2017; Liu et al., 2018; Wang et al., 2018), because the setup is simple and the outcome particle is almost uniform in size with high QY. In a typical approach, small organic molecules and/or polymers are dissolved in water or organic solvent to form the reaction precursor, which was then transferred to a Teflon-lined stainless steel autoclave. The organic molecules and/or polymers merged together at relatively high temperature to form carbon seeding cores and then grow into CQDs with a particle size of less 10 nm (Anwar et al., 2019). Zhu et al. (2013) reported the highest QY of CQDs up to about 80%, which is almost equal to fluorescent dyes. The CQDs were synthesized by using citric acid and ethylene diamine as carbon and nitrogen sources with high product yield under hydrothermal process, featuring as a desirable biosensor for the detection of Fe^3+^ in living cells. Hola et al. (2017) prepared full-color CQDs with controllable fluorescence at various wavelengths by tuning the amount of graphitic nitrogen under hydrothermal condition. Moreover, Lu et al. (2019) found that biomolecules with rich carbon and nitrogen resource can be used to finely tune the inner structures of CQDs under hydrothermal condensation. The facile synthetic process and controllable heteroatom doping make this method as promising approach to design and fabricate novel electrocatalyst with tunable doping composition and electronic structures.

### Electrochemistry Method

The electrochemical method is a simple and convenient preparation technique, which can be carried out under normal temperature and pressure conditions. Synthesis of CQDs by electrochemistry method has been widely reported for the sake that it is facile to tune the particle size and PL performance of the synthesized CQDs (Deng et al., 2014; Ahirwar et al., 2017; Anwar et al., 2019). In 2015, Hou et al. (2015) prepared a blue-emission CQDs with an averaged particle size of 2.4 nm by electrochemical carbonization of sodium citrate and urea in deionized (DI) water, which can be utilized as a highly sensitive detector for Hg^2+^ in waste water. Electrochemical synthesis method is also effective and widely used to fabricate efficient electrocatalyst, but for the CQDs synthesized by this method applied for electrocatalyst is rarely reported. Therefore, the integration of CQDs synthesis and electrocatalyst construction through one-pot electrochemical production is intriguing.

## Physical and Chemical Properties

### Absorbance

Generally, the optical absorption peaks of CQDs in the UV-visible region is usually estimated as π-π^*^ transition of sp^2^ conjugated carbon and n-π^*^ transition of hybridization with heteroatom such as N, S, P, etc. The absorption property can be manipulated through surface passivation or modification process (Zhao et al., 2015; Jiang et al., 2016; Li et al., 2018b; Anwar et al., 2019). Jiang et al. developed a facile hydrothermal method to synthesize red, green and blue luminescent CQDs by using three isomers of phenylenediamines (Jiang et al., 2015). The UV-visible absorption spectra of the as-obtained CQDs exhibited analogous pattern. Interestingly, the absorption transitions of these three CQDs were red-shifted, indicating the electronic bandgaps of the CQDs were smaller than their corresponding precursors.

### Photoluminescence

Photoluminescence is one of the most fascinating features of CQDs, both from the view of fundamental research and practical application (Peng and Travas-Sejdic, 2009; Gan et al., 2013; Lan et al., 2017; Li et al., 2018a; Yuan et al., 2018). In general, one uniform feature of the PL for CQDs is the distinct dependence of the emission wavelength and intensity. The reason for this unique phenomenon may be the optical selection of nanoparticles with different size or CQDs with different emissive traps on the surface (Li et al., 2012a). The variation of particle size and PL emission can be reflected from the broad and excitation-dependent PL emission spectrum (Sun et al., 2006). Zhang et al. (2017b) studied the emission behaviors of CQDs under an irradiation at 470 nm wavelength with various concentrations. It was found that the PL strength of the CQDs solution first increased and then decreased as the concentration increased.

### Electroluminescence

Since semiconductor nanocrystals are well-known to display electroluminescence (ECL), there should be no surprise that CQDs have inspired various interests for ECL studies which can favorably be used in electrochemical fields (Zhang et al., 2017a; Hasan et al., 2018; Xu et al., 2018). Zhang et al. (2013) reported a CQDs-based light-emitting diodes (LED) device, in which the emission color can be controlled by the driving current. Color-switchable ECL from the same CQDs ranging from blue to white was observed under different working voltages.

In order to understand the luminescence mechanism of CQDs more clearly, the researchers proposed two models based on the band gap emission of the conjugated p domain and the edge effect caused by another surface defect (Sk et al., 2014). The PL characteristics of the fluorescence emission of CQDs from the conjugated p domain are derived from the quantum confinement effect (QCE) of p-conjugated electrons in the sp^2^ atomic framework and can be adjusted by their size, edge configuration, and shape. Fluorescence emission of CQDs associated with surface defects results from sp^2^ and sp^3^ hybridized carbon and other surface defects of CQDs, and even fluorescence intensity and peak position are related to this defect (Shen et al., 2011; Zhu et al., 2012; Wang et al., 2014; Yuan et al., 2015).

## Application of Carbon Quantum Dots in Electrocatalysis

**Figure 2:**
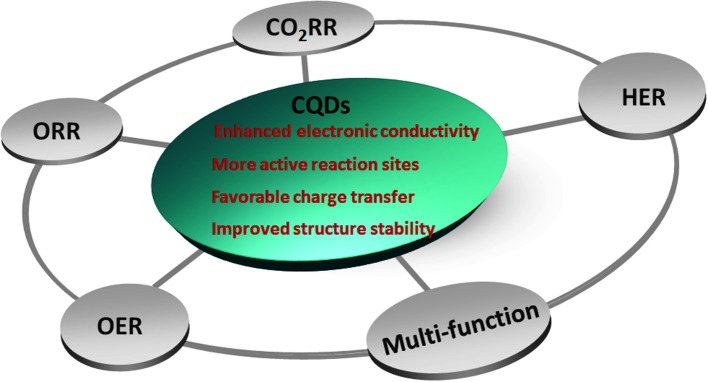
The electrocatalytic application of CQDs.

Carbon-based materials, especially CQDs, have gained plenty of interests in the fields of energy conversion and storage owing to the emerging tricky environmental issues (Lim et al., 2015). The abundant functional groups (-OH, -COOH, -NH_2_, etc.) on the surface of CQDs can be worked as active coordination site with transition metal ions. The heteroatom doped CQDs with multiple component may further enhance the electrocatalytic performance by promoting electron transfer via internal interactions. Particularly, CQDs hybridized with other inorganic compounds, such as layered-double-hydroxides (LDHs), metal sulfides, and metal phosphides, etc. can be utilized as efficient electrocatalysts for ORR, OER, HER, and CO_2_RR, etc. as shown in Figure 2, in view of the following reasons: (1) the cheap and easy accessibility of CQDs compared with the state-of-art precious metals; (2) the enhanced electronic conductivity of the hybrids stemming from CQDs; (3) more active catalytic reaction sites provided by CQDs; (4) favorable charge transfer during electrocatalytic process, and (5) the improved structure stability after bonding with CQDs.

### Oxygen Reduction Reaction

ORR, as a key route for metal-air batteries and fuel cells, have attracted much attention in recent years. The functional groups rich in nitrogen and oxygen make CQDs stable in water and several polar organic solvents, and provide convenience for multi-component photoelectric chemical reactions, including ORR (Li et al., 2012b; Fei et al., 2014; Zhang and Dai, 2015). Jin et al. developed a novel carbon-based ORR catalysts by hybridizing GQDs with graphene nanoribbons (GNR) through an *in situ* one-step reduction reaction. The obtained GQDs-GNR catalyst exhibited excellent performance and high durability in alkaline condition for ORR (Jin et al., 2015). Liu et al. fabricated a multidimensional N-GQDs hybrids with tunable PL emission by decorating a zero-dimension GQDs particle on the surface of two-dimension GQDs nanosheet. The thus-designed N-GQDs exhibited outstanding catalytic ORR performance in basic media (Liu and Wu, 2013). In 2015, a facile strategy was rationally designed by Xu et al. (2015) for the *in situ* reduction and assembly of GQDs with S-doped graphitic carbon nitride nanosheets (s-g-C_3_N_4_) under hydrothermal treatment. The resultant s-g-C_3_N_4_@GQDs nanohybrid exhibited significantly improved catalytic ORR activity compared with the pristine s-g-C_3_N_4_ and GQDs, indicating the promising potential of CQDs in electrocatalytic application.

### Oxygen Evolution Reaction

A combination of CQDs with transition metal (TM)-based compound could enhance the catalytic performance of TM-based catalyst (Mohanty et al., 2018; Wei et al., 2018). It has been reported that CoP/CQDs composite exhibits better OER activity with an overpotential of 400 mV in alkaline electrolytes than pure CoP (Zhu et al., 2018). The enhanced electrical catalytic performance of CoP/CQDs composite is due to the abundance of functional groups, small size, good conductivity and rapid electron transfer of CQDs. Tang et al. (2014) reported a promising CQDs/NiFe-LDH nanocomposite catalyst synthesized through a rational coprecipitation process, followed by a solvothermal treatment (Tang et al., 2014). The as-obtained CQDs/NiFe-LDH catalyst exhibited excellent OER electrochemical activity with low overpotential of ~235 mV at 10 mA cm^−2^ and good durability in 1 M KOH. The improved OER activity of CQDs/NiFe-LDH was attributed to the favorable electron transfer between CQDs and NiFe-LDH and the increased conductivity of NiFe-LDH.

### Hydrogen Evolution Reduction

In previous studies, CQDs-based composite nanomaterials have been proved to be potential electrocatalysts for energy conversion and storage. Because CQDs has good electric conductivity and sufficient active reaction sites, CQDs-based hybrid materials are successfully applied to electrocatalytic HER (Zhao et al., 2016; Wang et al., 2017; Zhang et al., 2018; Tian et al., 2019). In 2018, Li et al. (2018c) developed a facile thermal decomposition method for the fabrication of hybrid Ru@CQDs. The as-synthesized Ru@CQDs displayed excellent HER activity in alkaline conditions, i.e., it only requires an overpotential of 10 mV to achieve the current density of 10 mA cm^−2^ and shows a Tafel slope of 47 mV dec^−1^. Density function theory (DFT) calculation revealed the synergetic effect between Ru and CQDs, which is responsible for the high yield of Ru@CQDs and excellent catalytic HER activity. Tian et al. (2019) evaluated the HER activities of a class of advanced Ni_5_Mo_3_P@CQDs electrocatalysts. It was revealed that the CQDs not only modulate the morphology of the Ni_5_Mo_3_P composite, but also provide more reactive catalytic sites. Higher specific surface area can provide more electrochemical active sites and larger contact area with electrolyte, thus greatly promoting the HER performance. The as-obtained hollow Ni_5_Mo_3_P@CQDs nanosphere exhibited an excellent HER activity with lower overpotential, smaller Tafel slope, and favorable durability in acidic media compared with the single Ni_5_Mo_3_P.

### CO_2_ Reduction Reaction

Electrochemically converting the excess carbon dioxide produced in the combustion of fossil fuels back to the natural carbon cycle has become a hotspot of new energy research in recent years (Zou et al., 2017; Zhao et al., 2018; Gao et al., 2019). Fu and Zhu (2018) developed a novel strategy to fabricate nitrogen-doped graphitic quantum dots-wrapped single-crystalline Au nanoparticles (NGQDs-SCAu NPs) for the efficient CO_2_ reduction. The synthesized NGQDs-SCAu NPs displayed an enhanced performance toward CO_2_ reduction with an onset potential of 0.15 V vs. reversible hydrogen electrode (RHE) and an overpotential of only 0.04 V. Besides, the faradaic efficiency of the CO product of NGQDs-SCAu NPs was 0.4 V lower than that of the bare SCAu catalyst. The improved catalytic activity of NGQDs-SCAu NPs for reducing CO_2_ into CO was attributed to the synergetic effect between SCAu and NGQDs for the enhancement of COOH^*^ adsorption on the pyridine N site of NGQDs.

### Bifunctional Catalyst

CQDs can provide more catalytically active sites by both surrounding edges and many functional groups in electrocatalysis (Wei et al., 2018; Cirone et al., 2019; Shin et al., 2019; Zhang et al., 2019). Lv et al. (2017) prepared a bifunctional precious-metal-free electrocatalyst by *in-situ* formation of nitrogen-doped graphene quantum dots (NGQDs) and Ni_3_S_2_ nanocomposites on nickel foam (NF). The as-obtained Ni_3_S_2_-NGQDs/NF can be used as catalyst for overall water splitting with an overpotential of 216 mV for OER and 218 mV for HER to drive a current density of 10 mA cm^−2^ in alkaline media, separately. Furthermore, it required a cell voltage of 1.58 V to achieve 10 mA cm^−2^ in a two-electrode alkaline electrolyzer for overall water splitting. It was revealed that the synergetic effect between Ni_3_S_2_ and NGQDs played a critical role in the improvement of the electrocatalytic OER, HER, and overall water splitting performance of Ni_3_S_2_-NGQDs/NF nanocomposites. Tian et al. (2018) reported the fabrication of heterostructured nanosheet arrays of ternary nickel-cobalt phosphide (NiCo_2_P_2_) and GQDs supported on titanium mesh, which can act as the dual-function catalyst for both HER and OER. NiCo_2_P_2_/GQDs is more prominent than NiCo_2_O_4_/GQDs synthesized under the same condition and NiCo_2_P_2_ nanowires synthesized without GQDs. More importantly, NiCo_2_P_2_/GQDs outperformed the current commercial catalysts Pt/C/RuO_2_. The superior performance of NiCo_2_P_2_/GQDs are ascribed to the key role of GQDs in morphology modulation, enhanced electron transfer, and improved catalytic activity.

## Conclusions and Future Perspectives

The luminescent CQDs are interesting newcomers of nanomaterials, emerging more and more mature applications in the fields of chemical sensor, bioimaging, nanomedicine, drug delivery, and electrocatalysis. The main synthesis methods and photochemical properties of CQDs are introduced in this paper, and on this basis, its application in the field of electrocatalysis is emphatically addressed. A variety of synthesis techniques already developed for producing CQDs with different structures and characters are presented. The absorbance and PL properties of CQDs are both fascinating and intriguing, becoming as an active and hot research topic. However, the application of CQDs in electrocatalytic field is still in the infancy. More efforts on the novel design and fabrication of CQDs-based electrocatalyst are urgently needed.

For the synthesis of CQDs utilized for electrocatalyst, hydrothermal method is a promising candidate due to the facile controllable composition and structure via precursor optimization. Besides, electrochemical synthesis of CQDs is also a desirable alternative, which can produce CQDs with uniform particle size, and more importantly, it enable the cooperation of CQDs with other traditional electrocatalysts in one-pot production with a green chemistry process.

The large surface area, good conductivity, fast charge transfer of CQDs endow them with great potential for application in electrocatalysis. The unique electronic and chemical structures of CQDs can be adjusted by their size, shape, surface functional groups, and heteroatom doping. The rich organic groups enable the facile adsorption of water molecule and provide active coordinating sites with metal ions to form CQDs hybridized catalyst. The heteroatoms (i.e., N, S, P) doped in CQDs not only play a critical role in engineering the electronic structures of the adjacent carbon atoms within CQDs, but also act as reactive catalytic sites during the electrocatalytic process. Furthermore, CQDs could also protect the metal sites from poison and oxidation in the solution.

In the future, we expect the advent of more economic, facile and innovative synthetic methods and novel promising applications to better fulfill the potential of this increasingly significant carbon materials.

## Author Contributions

XW and YF organized and wrote the manuscript. PD and JH discussed the results. All the authors approved this manuscript.

### Conflict of Interest

The authors declare that the research was conducted in the absence of any commercial or financial relationships that could be construed as a potential conflict of interest.
